# Supplementary material for: Diffusion models for robotic manipulation: a survey
Source: Front Robot AI. 2025 Sep 9;12:1606247. doi: 10.3389/frobt.2025.1606247 (PMC12454101; doi:10.3389/frobt.2025.1606247)
Supplement: Supplementary file 1 [file Supplementaryfile1.pdf]

Supplementary Material

1 SUPPLEMENTARY TABLES

Encoder	Reference
**Vision**	
ResNet	He et al. (2016)
PointNet++	Qi et al. (2017)
Vision Transformer (ViT)	Dosovitskiy et al. (2020)
VQ-GAN	Esser et al. (2021)
OccNet	Mescheder et al. (2019)
VN-DGCNN	Deng et al. (2021)
Equivariant U-Net	Ryu et al. (2023)
VN-PointNet	Deng et al. (2021)
BPS	Prokudin et al. (2019)
ShapeEncoder	Park et al. (2019)
**Vision-Language**	
CLIP	Radford et al. (2021)
SAM	Kirillov et al. (2023)
XMem	Cheng and Schwing (2022)
HULC	Mees et al. (2022a)
T5	Raffel et al. (2020)

Table S1. References for architectures of encoders, for different input modalities.

Dataset	Reference
**Trajectories**	
Adroit	Fu et al. (2020)
BEHAVIOR	Srivastava et al. (2022)
BridgeData	Walke et al. (2023)
CALVIN	Mees et al. (2022b)
D3IL	Jia et al. (2024)
D4RLKitchen	Fu et al. (2020)
DexDeform	Ma et al. (2024)
EpicKitchens	Damen et al. (2021)
Fetch env	Ma et al. (2022)
FrankaKitchen	Gupta et al. (2020)
FurnitureBench	Heo et al. (0)
KUKA	Diffuser
LabGym	Maria Scheikl et al. (2023)
LIBERO	Liu et al. (2023)
MetaWorld	Yu et al. (2020)
MπNets	Fishman et al. (2023)
STAP	Agia et al. (2022)
Ravens	Zeng et al. (2021); Shridhar et al. (2022)
Relay Kitchen	Gupta et al. (2020)
RLBench	James et al. (2020)
Robomimic	Mandlekar et al. (2022)
RT1	Brohan et al. (2023)
XArm Block Push	Florence et al. (2022)
**Grasps**	
Acronym	Eppner et al. (2021)
DA^2	Zhai et al. (2022)
DexGraspNet	Wang et al. (2023)
MultiDex	Li et al. (2024)
OakInk	Yang et al. (2022)
VGN	Breyer et al. (2021)

Table S2. List of datasets and their corresponding references for trajectory diffusion and grasp diffusion.

REFERENCES

Agia, C., Migimatsu, T., Wu, J., and Bohg, J. (2022). Taps: Task-agnostic policy sequencing. *arXiv preprint arXiv:2210.12250*

Breyer, M., Chung, J. J., Ott, L., Siegwart, R., and Nieto, J. (2021). Volumetric grasping network: Real-time 6 dof grasp detection in clutter. *Conference on Robot Learning* , 1602–1611

Brohan, A., Brown, N., Carbajal, J., Chebotar, Y., Dabis, J., Finn, C., et al. (2023). RT-1: Robotics transformer for real-world control at scale. *arXiv preprint arXiv:2212.06817*

Cheng, H. K. and Schwing, A. G. (2022). Xmem: Long-term video object segmentation with an atkinson-shiffrin memory model. *Computer Vision – ECCV 2022* , 640–658

Damen, D., Doughty, H., Farinella, G. M., Fidler, S., Furnari, A., Kazakos, E., et al. (2021). The EPIC-KITCHENS Dataset: Collection, Challenges and Baselines. *IEEE Transactions on Pattern Analysis and Machine Intelligence (TPAMI)* 43, 4125–4141. doi:10.1109/TPAMI.2020.2991965

Deng, C., Litany, O., Duan, Y., Poulenard, A., Tagliasacchi, A., and Guibas, L. J. (2021). Vector neurons: A general framework for so (3)-equivariant networks. *Proceedings of the IEEE/CVF International Conference on Computer Vision* , 12200–12209

Dosovitskiy, A., Beyer, L., Kolesnikov, A., Weissenborn, D., Zhai, X., Unterthiner, T., et al. (2020). An Image is Worth 16x16 Words: Transformers for Image Recognition at Scale. *International Conference on Learning Representations*

Eppner, C., Mousavian, A., and Fox, D. (2021). Acronym: A large-scale grasp dataset based on simulation. *2021 IEEE International Conference on Robotics and Automation (ICRA)* , 6222–6227

Esser, P., Rombach, R., and Ommer, B. (2021). Taming Transformers for High-Resolution Image Synthesis. *2021 IEEE/CVF Conference on Computer Vision and Pattern Recognition (CVPR)* , 12868–12878doi:10.1109/CVPR46437.2021.01268

Fishman, A., Murali, A., Eppner, C., Peele, B., Boots, B., and Fox, D. (2023). Motion Policy Networks. *Proceedings of The 6th Conference on Robot Learning* 205, 967–977

Florence, P., Lynch, C., Zeng, A., Ramirez, O., Wahid, A., Downs, L., et al. (2022). Implicit Behavioral Cloning. *Proceedings of Machine Learning Research* 164, 158–168

Fu, J., Kumar, A., Nachum, O., Tucker, G., and Levine, S. (2020). D4RL: Datasets for Deep Data-Driven Reinforcement Learning. *arXiv preprint arXiv:2004.07219*

Gupta, A., Kumar, V., Lynch, C., Levine, S., and Hausman, K. (2020). Relay Policy Learning: Solving Long-Horizon Tasks via Imitation and Reinforcement Learning. *Proceedings of Machine Learning Research* , 1025–1037

He, K., Zhang, X., Ren, S., and Sun, J. (2016). Deep Residual Learning for Image Recognition. *Proceedings of the IEEE Conference on Computer Vision and Pattern Recognition (CVPR)*

Heo, M., Lee, Y., Lee, D., and Lim, J. J. (0). FurnitureBench: Reproducible real-world benchmark for long-horizon complex manipulation. *The International Journal of Robotics Research* 0, 02783649241304789. doi:10.1177/02783649241304789

James, S., Ma, Z., Arrojo, D. R., and Davison, A. J. (2020). RLBench: The Robot Learning Benchmark & Learning Environment. *IEEE Robotics and Automation Letters* 5, 3019–3026. doi:10.1109/LRA.2020.2974707

Jia, X., Blessing, D., Jiang, X., Reuss, M., Donat, A., Lioutikov, R., et al. (2024). Towards Diverse Behaviors: A Benchmark for Imitation Learning with Human Demonstrations. *The Twelfth International Conference on Learning Representations, ICLR 2024, Vienna, Austria, May 7-11, 2024*

Kirillov, A., Mintun, E., Ravi, N., Mao, H., Rolland, C., Gustafson, L., et al. (2023). Segment Anything. *Proceedings of the IEEE/CVF International Conference on Computer Vision (ICCV)* , 4015–4026

Li, P., Wang, Z., Liu, M., Liu, H., and Chen, C. (2024). ClickDiff: Click to Induce Semantic Contact Map for Controllable Grasp Generation with Diffusion Models. *Proceedings of the 32nd ACM International Conference on Multimedia* , 273–281doi:10.1145/3664647.3680597

Liu, B., Zhu, Y., Gao, C., Feng, Y., Liu, Q., Zhu, Y., et al. (2023). LIBERO: Benchmarking Knowledge Transfer for Lifelong Robot Learning. *Advances in Neural Information Processing Systems* 36, 44776–44791

Ma, C., Yang, H., Zhang, H., Liu, Z., Zhao, C., Tang, J., et al. (2024). DexDiff: Towards Extrinsic Dexterity Manipulation of Ungraspable Objects in Unrestricted Environments. *arXiv preprint arXiv:2409.05493*

Ma, J. Y., Yan, J., Jayaraman, D., and Bastani, O. (2022). Offline Goal-Conditioned Reinforcement Learning via f-Advantage Regression. *Advances in Neural Information Processing Systems* 35, 310–323

Mandlekar, A., Xu, D., Wong, J., Nasiriany, S., Wang, C., Kulkarni, R., et al. (2022). What Matters in Learning from Offline Human Demonstrations for Robot Manipulation. *Proceedings of the 5th Conference on Robot Learning* 164, 1678–1690

Maria Scheikl, P., Gyenes, B., Younis, R., Haas, C., Neumann, G., Wagner, M., et al. (2023). LapGym-An Open Source Framework for Reinforcement Learning in Robot-Assisted Laparoscopic Surgery. *Journal of Machine Learning Research* 24, 1–42

Mees, O., Hermann, L., and Burgard, W. (2022a). What Matters in Language Conditioned Robotic Imitation Learning Over Unstructured Data. *IEEE Robotics and Automation Letters* 7, 11205–11212. doi:10.1109/LRA.2022.3196123

Mees, O., Hermann, L., Rosete-Beas, E., and Burgard, W. B. (2022b). CALVIN: A Benchmark for Language-Conditioned Policy Learning for Long-Horizon Robot Manipulation Tasks. *IEEE Robotics and Automation Letters* 7, 7327–7334. doi:10.1109/LRA.2022.3180108

Mescheder, L., Oechsle, M., Niemeyer, M., Nowozin, S., and Geiger, A. (2019). Occupancy networks: Learning 3d reconstruction in function space. *Proceedings of the IEEE/CVF conference on computer vision and pattern recognition* , 4460–4470

Park, J. J., Florence, P., Straub, J., Newcombe, R., and Lovegrove, S. (2019). Deepsdf: Learning continuous signed distance functions for shape representation. *Proceedings of the IEEE/CVF conference on computer vision and pattern recognition* , 165–174

Prokudin, S., Lassner, C., and Romero, J. (2019). Efficient learning on point clouds with basis point sets. *Proceedings of the IEEE/CVF international conference on computer vision* , 4332–4341

Qi, C. R., Yi, L., Su, H., and Guibas, L. J. (2017). Pointnet++: Deep hierarchical feature learning on point sets in a metric space. *Advances in neural information processing systems* 30

Radford, A., Kim, J. W., Hallacy, C., Ramesh, A., Goh, G., Agarwal, S., et al. (2021). Learning transferable visual models from natural language supervision. *International conference on machine learning* , 8748–8763

Raffel, C., Shazeer, N., Roberts, A., Lee, K., Narang, S., Matena, M., et al. (2020). Exploring the Limits of Transfer Learning with a Unified Text-to-Text Transformer. *Journal of Machine Learning Research* 21, 1–67

Ryu, H., in Lee, H., Lee, J.-H., and Choi, J. (2023). Equivariant Descriptor Fields: SE(3)-equivariant energy-based models for end-to-end visual robotic manipulation learning. *The Eleventh International Conference on Learning Representations*

Shridhar, M., Manuelli, L., and Fox, D. (2022). CLIPort: What and Where Pathways for Robotic Manipulation. *Proceedings of the 5th Conference on Robot Learning* 164, 894–906

Srivastava, S., Li, C., Lingelbach, M., Martín-Martín, R., Xia, F., Vainio, K. E., et al. (2022). BEHAVIOR: Benchmark for Everyday Household Activities in Virtual, Interactive, and Ecological Environments. *Proceedings of the 5th Conference on Robot Learning* 164, 477–490

Walke, H. R., Black, K., Zhao, T. Z., Vuong, Q., Zheng, C., Hansen-Estruch, P., et al. (2023). BridgeData V2: A Dataset for Robot Learning at Scale. *Proceedings of The 7th Conference on Robot Learning* 229, 1723–1736

Wang, R., Zhang, J., Chen, J., Xu, Y., Li, P., Liu, T., et al. (2023). Dexgraspnet: A large-scale robotic dexterous grasp dataset for general objects based on simulation. *2023 IEEE International Conference on Robotics and Automation (ICRA)* , 11359–11366

Yang, L., Li, K., Zhan, X., Wu, F., Xu, A., Liu, L., et al. (2022). Oakink: A large-scale knowledge repository for understanding hand-object interaction. *Proceedings of the IEEE/CVF conference on computer vision and pattern recognition* , 20953–20962

Yu, T., Quillen, D., He, Z., Julian, R., Hausman, K., Finn, C., et al. (2020). Meta-World: A Benchmark and Evaluation for Multi-Task and Meta Reinforcement Learning. *Proceedings of the Conference on Robot Learning* 100, 1094–1100

Zeng, A., Florence, P., Tompson, J., Welker, S., Chien, J., Attarian, M., et al. (2021). Transporter Networks: Rearranging the Visual World for Robotic Manipulation. *Proceedings of the 2020 Conference on Robot Learning* 155, 726–747

Zhai, G., Zheng, Y., Xu, Z., Kong, X., Liu, Y., Busam, B., et al. (2022). DA^2 Dataset: Toward Dexterity-Aware Dual-Arm Grasping. *IEEE Robotics and Automation Letters* 7, 8941–8948
